# Diagnostic Biomarkers for Alzheimer’s Disease Using Non-Invasive Specimens

**DOI:** 10.3390/jcm9061673

**Published:** 2020-06-01

**Authors:** Maria Paraskevaidi, David Allsop, Salman Karim, Francis L. Martin, StJohn Crean

**Affiliations:** 1 School of Pharmacy and Biomedical Sciences, University of Central Lancashire, Preston PR1 2HE, UK; flmartin@uclan.ac.uk (F.L.M.); screan@uclan.ac.uk (S.C.); 2 Department of Metabolism, Digestion and Reproduction, Faculty of Medicine, Imperial College London, London W12 0NN, UK; 3 Division of Biomedical and Life Sciences, Faculty of Health and Medicine, Lancaster University, Lancaster LA1 4YW, UK; d.allsop@lancaster.ac.uk; 4 Central Lancashire Memory Assessment Service, Lancashire Care NHS Foundation Trust, Bamber Bridge, Preston PR5 6YA, UK; salman.karim@lancashirecare.nhs.uk

**Keywords:** Alzheimer’s disease, diagnostics, biomarkers, early detection, salivary markers, buccal cells

## Abstract

Studies in the field of Alzheimer’s disease (AD) have shown the emergence of biomarkers in biologic fluids that hold great promise for the diagnosis of the disease. A diagnosis of AD at a presymptomatic or early stage may be the key for a successful treatment, with clinical trials currently investigating this. It is anticipated that preventative and therapeutic strategies may be stage-dependent, which means that they have a better chance of success at a very early stage—before critical neurons are lost. Several studies have been investigating the use of cerebrospinal fluid (CSF) and blood as clinical samples for the detection of AD with a number of established core markers, such as amyloid beta (Aβ), total tau (T-tau) and phosphorylated tau (*P*-tau), being at the center of clinical research interest. The use of oral samples—including saliva and buccal mucosal cells—falls under one of the least-investigated areas in AD diagnosis. Such samples have great potential to provide a completely non-invasive alternative to current CSF and blood sampling procedures. The present work is a thorough review of the results and analytical approaches, including proteomics, metabolomics, spectroscopy and microbiome analyses that have been used for the study and detection of AD using salivary samples and buccal cells. With a few exceptions, most of the studies utilizing oral samples were performed in small cohorts, which in combination with the existence of contradictory results render it difficult to come to a definitive conclusion on the value of oral markers. Proteins such as Aβ, T-tau and *P*-tau, as well as small metabolites, were detected in saliva and have shown some potential as future AD diagnostics. Future large-cohort studies and standardization of sample preparation and (pre-)analytical factors are necessary to determine the use of these non-invasive samples as a diagnostic tool for AD.

## 1. Introduction

With a rapidly aging population across the world, Alzheimer’s disease (AD) is expected to affect 75 million people in 2030 with much of the increase happening in developing countries [1]. It is now recognized that pathologic changes in the brain commence several years before any obvious symptoms of memory loss [2,3] with amyloid beta (Aβ) pathology thought to be a key initial step, followed by the development of neurofibrillary tau pathology [4]. Three different stages were suggested to characterize the disease: preclinical (or asymptomatic) AD, mild cognitive impairment (MCI) due to AD and dementia due to AD [5]. An accurate premorbid diagnosis of the disease remains challenging and is currently based upon clinical presentations, as well as other imaging and biofluid biomarkers [6]. Positron emission tomography (PET) scans can reveal Aβ or tau accumulation in the brain whereas magnetic resonance imaging (MRI) can measure function and reveal brain atrophy [3]. Specific cerebrospinal (CSF) biofluid constituents, such as amyloid beta 42 (Aβ42), which correlates with extracellular senile plaques [7], total tau (T-tau), which reflects the intensity of neuronal damage [8] and phosphorylated tau (*P*-tau), which correlates with tangle pathology [8], were shown to have excellent diagnostic accuracy [9]. However, the relatively invasive nature of CSF collection limits its widespread use in routine primary care practice as the procedure is only rarely performed by general practitioners [10]. Moreover, a lumbar puncture can be unpleasant and generate patient anxiety and therefore is not preferred as a routine test.

Emerging evidence over the last decade supports the use of blood-based biomarkers for investigating AD [11,12,13,14,15]. Depending on its intended use, an effective biomarker can serve different purposes and have different characteristics. For instance, an ideal diagnostic marker would allow a highly accurate point-of-care in vivo test with high sensitivity and specificity, whereas a screening marker would combine low cost and favor sensitivity over specificity with further, more specific, follow-up tests needed. In contrast to CSF, blood sampling could provide an ideal cost-effective and relatively minimally invasive alternative sample for testing and has shown much promise over time for AD diagnosis, screening, prognosis and disease monitoring. The huge benefit of using blood samples comes from their potential to reveal AD-related changes at a presymptomatic or early stage of the disease [16,17]. With the emergence of promising drug candidates for the treatment of AD [18], it is anticipated that an early diagnosis is likely to define the course of the disease as administration of a candidate treatment is expected to be most effective if provided as early as possible [10,19,20,21] (i.e., before severe neuronal loss). Clinical trials assessing promising drug agents in individuals diagnosed with mild cognitive impairment (MCI) or early stage AD are currently ongoing (BAN2401: [22] and aducanumab: [23]).

Work in the area of dementia using CSF and blood samples is fairly advanced with many studies demonstrating useful differences between healthy controls and AD patients [9,15,17,24,25]. An overview of fluid biomarkers that have been replicated by different studies are reported in Table 1 [25].

A more recent and admittedly less explored, area of interest is the use of saliva and oral buccal mucosal cells as a means of diagnosing, monitoring and potentially screening for AD. This field has attracted more attention within the clinical and scientific community in recent years due to the successful advent of minimally invasive blood diagnostics as well as accumulating evidence linking AD with “oral-cavity arising” biomarkers. For instance, several studies have correlated AD development with oral bacteria and chronic periodontal disease [26,27,28]. It was hypothesized that oral bacteria or their released endotoxins, may access the brain and induce microglial activation, which is a well-known AD marker resulting in increased levels of proinflammatory cytokines, capable of initiating neuronal loss and neurodegeneration [29].

Overall, several studies have assessed non-invasive, oral-derived biomarkers towards AD diagnosis and this review will focus on the use of saliva and oral mucosa derived buccal cells, the most popular sources of oral samples [30]. Herein, we present an overview of the literature across the field, including potential diagnostic markers, promising analytical approaches as well as challenges and limitations during sample collection and processing.

## 2. Saliva

### 2.1. Saliva: General Considerations

Being one of the simplest and easily accessible non-invasive biofluids, saliva has drawn great attention as an ideal candidate for disease detection. Traditionally, salivary markers have been utilized in diagnostic and monitoring tests for periodontal and other oral diseases [31,32]. More recent technological advances, leading to an advent of sensitive analytical approaches and sensors, have created the attractive opportunity for the diagnosis of other systemic diseases using saliva, introducing a field of “salivary diagnostics” [33,34]. Previous studies have demonstrated the promise of salivary specimens in diagnosing tumors [35,36,37], viral infections [38], gastrointestinal diseases [39], diabetes [40,41] and muscle/joint disorders [33]. Salivary biomarkers have also emerged as a means of diagnosing neurodegenerative conditions such as AD [42], Parkinson’s disease [43] and multiple sclerosis [44].

A number of different hypotheses have been suggested over the years to explain the origin of salivary biomarkers indicative of AD. For instance, it has been proposed that biomarkers may be secreted by nerves into salivary glands due to their close proximity to the central nervous system [42]; or that salivary proteins originate after transport of molecules from blood to saliva through ultrafiltration and passive diffusion or active transport [45]. It is accepted that potential biomarkers reflecting brain pathology can be released at small concentrations in the blood after crossing the blood–brain barrier [46], which in the case of neurodegenerative disease is disrupted, thus leading to increased permeability [47]. Moreover, ~500 mL CSF is absorbed daily into the blood circulation, which makes blood an information-rich sample [48]. More recent studies have identified oral bacteria, capable of causing chronic inflammation, as potential culprits behind AD and this could be another plausible reason for the detection of salivary changes in AD affected individuals [26,27,49].

In comparison to the more-widely used blood specimens, saliva has some unique advantages, such as relative ease of collection (no experienced personnel is required creating the exciting possibility for self-collected samples) and completely non-invasive and inexpensive sample collection (no anticoagulants needed, relatively easier storage under the human tissue authority guidelines and permissions). Some challenges that need to be addressed in the use of saliva include the decreased concentration of analytes [50], which necessitates more sensitive analytical approaches, as well as the inability of approximately 1/3 of participants to produce an adequate saliva sample [51].

### 2.2. Saliva: Studies on AD

We identified 20 studies that reported the use of saliva as the sample of choice to study AD (Table 2). Considering the multifactorial nature of the disease itself, it is not surprising that different research groups have focused on the study of different biomarkers, both in isolation and as a panel of markers, as well as different experimental techniques. Overall, some biomarkers and experimental approaches have been studied more extensively than others, however, results are, in some cases, contradictory between the different studies. In this section, we will present an overview of the main findings of these studies.

### 2.3. Amyloid Beta (Aβ42 and/or Aβ40)

Most the included salivary studies (7/20) focused on the detection of the amyloid beta 42 (Aβ42) peptide, with a smaller number (2/20) also focusing on the Aβ40 peptide. All of the studies used an enzyme-like immunosorbent assay (ELISA) as their main experimental approach to detect Aβ. Most of the studies (5/7) demonstrated increased levels of Aβ42 in AD patients [52,53,54,55,56] while the others (2/7) showed undetectable or unchanged Aβ42 levels [42,57]. With the exception of a relatively larger study by Bermejo-Pareja et al. [55], including 70 AD patients and 56 healthy volunteers serving as the study’s controls, the remaining studies reported results on much smaller cohorts ranging from 7–28 AD patients and 7–38 healthy controls.

More specifically, Sabbagh et al. [52] recruited 15 AD patients (7 males, mean age: 77.8  years, mean mini-mental state examination (MMSE) score 19.0) and 7 normal controls (2 males, mean age: 60.4 years, mean MMSE 29.0) and demonstrated significantly higher salivary Aβ42 in AD patients than in controls (51.7 ± 1.6 pg/mL for AD and 21.1 ± 0.3 pg/mL for controls, *p* < 0.001). Participants were enrolled as mild and moderate AD according to the AD criteria established by the National Institute on Aging and the Alzheimer’s Association (NIA-AA) [79]. Inclusion criteria were MMSE scores of 10–26 and age ≥50 years. Healthy patients with normal cognitive functioning and no neurodegenerative disease who were intact functionally, physically and socially and were ≥50 years served as the control group for this study.

McGeer et al. [53] employed 23 AD individuals (8 males, mean age: 71.3 years) and 31 heathy controls (25 low-risk controls: 17 male, mean age: 54.2 years; and 6 high-risk controls: 3 males, mean age: 69.0 years). However, it was not clarified whether diagnosis of AD cases was based on clinical criteria and/or established biomarkers. Based on their findings, low-risk healthy controls had Aβ42 levels of ~20 pg/mL while high-risk controls and AD patients had increased Aβ42 levels ranging from 40–85 pg/mL (Aβ42 levels: AD > high-risk controls > low-risk controls). The authors concluded that such an approach would be useful in the identification of those at risk at an age well below the typical age of AD onset, thus reducing the prevalence of AD.

Lee et al. [54] determined salivary Aβ42 levels after treating the sample with thioflavin S as an anti-aggregation agent and sodium azide as an antibacterial agent. Utilizing a total of 10 AD/pre-AD cases (7 AD and 3 pre-AD) (3 males, mean age; 70.1) and 27 controls (including 1 Parkinson’s disease case) (18 males, mean age: 54.6), the authors demonstrated a twofold increase in Aβ42 concentration in the AD group when compared to controls, supporting its use in the diagnosis and potentially screening of AD. It was not clear in the study whether AD diagnosis was based on standard clinical criteria and/or established biomarkers.

Lau et al. [57] attempted to quantify Aβ42 levels using ELISA in 20 AD (8 male, mean age: 72.5, mean MMSE score 18.1) and 20 healthy control samples (9 males, mean age: 66.1, mean MMSE score 28.7), however this specific biomarker was not detected in the saliva. All participants underwent a series of clinical and neuropsychological tests (MMSE and the Clinical dementia Rating-Sum of Boxes (CDR-SOB)) and the control group consisted of volunteers ≥50 years of age without a history of neurological, psychiatric or other medical diagnosis that could contribute to cognitive impairment or dementia. The CDR-SOB rating was 6.2 for the AD group and 0.2 for the controls. The same study attempted measurement of other biomarkers indicative of AD, such as tau, results which will be provided in the relevant sub-sections below.

In a study by Bermejo-Pareja et al. [55], an increase was observed in the Aβ42 levels of mild (7.67 pg/mL) and moderate AD (11.70 pg/mL), but interestingly not in severe AD (3.03 pg/mL) when compared with healthy controls (2.89 pg/mL). The cohort consisted of 70 AD (29 mild AD, 24 moderate AD and 17 severe AD) (21 males, mean age: 77.2, mean MMSE score 17) and 56 controls (17 males, mean age: 74.4, mean MMSE score was not determined). All AD cases were diagnosed according to the Diagnostic and statistical manual of mental disorders (DSM)-IV and NINCDS–ADRDA criteria and diagnosis required evidence of cognitive decline as well as impairment in social or occupational function. All participants had extensive biochemical measurements and neuroimaging tests (brain MRI and/or CT scan). Classification of mild, moderate and sever AD was performed and the diagnosis of vascular dementia was excluded in all cases, using DSM-III-R criteria. The control group was formed of family members or caregivers of the AD patients after a clinical interview with a senior neurologist that showed a completely normal cognitive and functional level. No formal neuropsychological battery was performed in the control group. The association that was found between Aβ42 and AD was independent of established risk factors, including age or apolipoprotein E (ApoE) genotype, but was dependent on sex and functional capacity. This study also analyzed the Aβ_40_ levels which were found unchanged between AD patients and healthy subjects. The Aβ42/Aβ40 ratio was higher, but not statistically significant in mild and moderate AD patients, whereas it was unchanged in severe AD patients.

Kim et al. [56] reported a statistically significant increase in the salivary Aβ42 levels, however there was no statistical significance in Aβ40. This study included 28 AD (mean MMSE: 17.3) and 17 healthy control samples (mean MMSE: 25.5) and was capable of detecting Aβ peptides at concentrations as low as ~20 pg/mL. AD diagnosis was performed according to the clinical mental examination and on the basis of cognitive dysfunction, interference in daily life and intellectual decline. MMSE scoring was used as an indication of the cognitive function of patients and facilitate diagnosis. The control group was composed of volunteers with no family history of AD and a normal cognitive function and intellectual ability.

Shi et al. [42] involved 21 AD patients (10 male, mean age: 68.8, mean MMSE score 19.2) and 38 healthy volunteers (19 males, mean age: 69, mean MMSE score 29.4) in their study of saliva, showing that Aβ42 was not detectable using a sensitive ELISA approach. The authors also compared the salivary tau levels between AD and controls (more details in the relevant sub-section). The clinical diagnosis of probable AD was made using the National Institute of Neurological, Communicative Disorders and Stroke–Alzheimer’s disease and Related Disorders Association (NINCDS–ADRDA) criteria while all control subjects were community volunteers with a MMSE score ≥27, paragraph recall scores >6, no history of neurological disease and no history or evidence of cognitive or functional decline.

### 2.4. Tau (T-tau and/or P-tau)

We identified four studies of salivary tau biomarkers, either in combination with other markers or in isolation. None of these studies found statistically significant changes in the levels of total tau (T-tau) or phosphorylated tau (*P*-tau) while two studies showed consistent findings with an increased *P*-tau/T-tau ratio (i.e., the proportion of the phosphorylated isoform of tau).

The Lau et al. study [57], apart from Aβ42, also studied tau protein levels in 20 AD and 20 healthy control and found no significant differences in T-tau or *P*-tau. Details on the diagnosis of AD cases were provided in the previous section (Amyloid beta). A separate metabolite (the salivary sugar trehalose) was found, however, to distinguish between the two groups in the same study (more details in the ‘Metabolites’ sub-section).

Shi et al. [42], studying 21 AD patients and 38 healthy volunteers, showed no significant change in T-tau or *P*-tau between the cohorts whereas the ratio *P*-tau/T-tau was significantly increased in the AD group. Details on the diagnosis of AD cases were provided in the previous section.

Ashton et al. [58] was the first large-cohort study of its kind to employ the ultrasensitive single molecule array (SIMOA) to measure T-tau in saliva. The cohort consisted of 53 AD patients (23 males, mean age: 81.4) 68 individuals with mild cognitive impairment (MCI) (33 males, mean age: 79.8) and 160 healthy controls (66 males, mean age: 78.0) and final results showed no statistically significant differences across these diagnostic groups, confirming the preliminary findings of the other two studies. The diagnosis of probable AD was made according to Diagnostic and Statistical Manual for Mental Diagnosis, fourth edition and NINCDS–ADRDA criteria [80]. MCI was defined according to Petersen criteria [81]. Standardized clinical assessment included the MMSE and for global levels of severity the Clinical Dementia Rating. The AD and MCI groups also had MRI scans.

Pekeles et al. [59] utilized western blot analysis to quantify the P-tau/T-tau ratio of a total of 87 AD patients, 55 MCI and 167 healthy controls. For technical reasons, the analysis was carried out in two rounds, the first with 148 samples including 46 AD (24 males, mean age: 80.0), 55 MCI (23 males, mean age: 78.0) and 47 control subjects (15 males, mean age: 73.0) and the second with a total of 189 samples including 41 AD (17 males, mean age: 80.0), 44 controls (14 males, mean age: 72.0), 16 frontotemporal dementia (FTD) (11 males, mean age: 71.5), 12 neurology (i.e., Individuals with brain diseases such as stroke, epilepsy and multiple sclerosis, but normal cognitive function) (5 males, mean age: 55.0) and 76 young normal subjects (31 males, mean age: 32.0). Probable AD diagnosis was given according to the McKhann et al. criteria for dementia [79], including significant cognitive impairment and symptoms sufficient to interfere with work or daily activities, a gradual onset of symptoms, with either deficits in learning and recall or language, visuospatial or executive problems and absence of other neurological diseases causing the symptoms. Evidence of pathophysiological processes, such as neurofibrillary tangles or amyloid plaques further increased the certainty of diagnosis. Diagnosis of MCI was made if the subject displayed subjective memory complaints, had normal activities of daily living and general cognitive function, and demonstrated objective evidence of mild memory impairment on testing. Healthy controls were volunteers aged ≥60 years and screened with the Montreal Cognitive Assessment (MoCA), which had to be ≥25. The authors demonstrated a significant elevation of *P*-tau/T-tau ratio, however large variations in the AD salivary levels limits the utility of the test as a clinical biomarker. Moreover, the observed elevation in saliva did not correlate with CSF tau or with brain measures, such as hippocampal volume.

### 2.5. Lactoferrin

Other biomarkers, apart from Aβ and tau proteins, have also shown potential as diagnostic means in saliva specimens. For instance, lactoferrin, an antimicrobial peptide with a known Aβ-binding ability, was found to be significantly reduced in AD patients in comparison to healthy controls [60]. Carro et al. [60] measured the salivary lactoferrin concentration in two separate studies, one being the discovery and the other being the validation study. The discovery study utilized an initial mass spectrometry approach and consisted of 80 AD individuals (31 males, mean age: 76.2), 44 MCI (19 males, mean age: 75.2), 91 healthy controls (32 males, mean age: 73.7) and 69 Parkinson’s disease patients, while the validation study utilized an ELISA assay and consisted of 36 AD patients (12 males, mean age: 80.7), 15 MCI (10 males, mean age: 68.3) and 40 healthy controls (15 males, mean age: 66.8). AD diagnosis was established according to the NINCDS–ADRDA guidelines [79] while MCI according to the Petersen criteria [81]. Inclusion criteria for cognitively normal older individuals were MMSE scores of 29.0, no history or clinical signs of neurological or psychiatric disease or cognitive symptoms. Lactoferrin was positively correlated with MMSE score and Aβ42 while negatively correlated with T-tau. It was also demonstrated that 14 out of 18 controls that were found with abnormally reduced lactoferrin levels (<7.43 μg/mL) had converted to a clinical diagnosis of MCI or AD over the course of the study (1–5 years), whereas that was not the case for subjects with normal/high levels of lactoferrin (≥7.43 μg/mL). The authors concluded that this marker may prove promising in population screening and in identifying underdiagnosed individuals with very early stages of MCI and AD.

### 2.6. Acetylcholinesterase Activity

Three separate studies were identified to measure acetylcholinesterase activity in saliva using the Ellman colorimetric method [61,62,63]. Acetylcholinesterase (AchE) is an enzyme that degrades the neurotransmitter acetylcholine (Ach) and therefore limits its postsynaptic effects. Acetylcholinesterase inhibitors (AchE-I) are the primary drug prescribed in AD patients for symptom management, which encourages an increase in and prolonged activity period for the released Ach due to the inhibition of AchE. It was previously established that AD is linked with a deficiency in the brain neurotransmitter ACh due to cholinergic neuron degradation. These same neurons synthesize and release the ACh limiting AchE and thus levels of AChE are indicative of cholinergic neuron health. Several studies have attempted to measure its activity in saliva.

Sayer et al. [61] recruited a total of 36 AD patients (22 AD responders to AchE-I (7 males, men age: 75.0) and 14 AD nonresponders (4 males, mean age: 75.0)) and 11 healthy controls (6 males, mean age: 71.0). Subjects were diagnosed using McKhann et al. criteria [79], supplemented by information from an informed caregiver and MMSE scores. Control subjects were relatives, caregivers or friends of patients attending the clinic. None of the control subjects had evidence of any psychiatric syndrome and all controls made no errors on the MMSE. This study reported a decreased activity of salivary AchE in people with AD when compared to age-matched controls. In addition, a significant age-related decrease was found in the catalytical activity of the enzyme in a control cohort, which reflected the age-association in cholinergic function. In addition, AchE was significantly decreased in the AchE-I nonresponder group when compared to the responder group.

A later study by Boston et al. [62], with 15 AD patients (5 males, mean age: 83.5, mean MMSE score 20.4), 13 healthy controls (7 males, mean age: 80.8, mean MMSE score 29.1) and 13 vascular dementia patients (9 males, mean age: 81.8, mean MMSE score 18.4), found a decreasing trend in the activity of AChE within the AD cohort, however the differences were not statistically significant. Diagnosis of AD was given according to NINCDS–ADRDA criteria while control subjects were individuals without dementia or significant physical illness, mainly from the relatives of the patients who entered the study.

Bakhtiari et al. [63] employed 15 AD (9 males, mean age: 78.4) and 15 healthy controls (7 males, mean age: 71.0) and showed that although there was a decreased AChE activity in AD, there was no statistically significant difference (*p* value = 0.25). AD patients were previously diagnosed and were on memantine therapy while the control group consisted of randomly selected elderly non-demented subjects without neurological or cognitive disease.

The authors of the 2 later studies [62,63] demonstrating no change in the AChE activity hypothesized that the observed decrease in the Sayer study [61] may be due to the treatment with AChE-I that created long term adaptive changes in the production of AchE.

### 2.7. Oral Microbiome

More recent studies have focused on the oral microbiome as a diagnostic means for AD [26,64], however these have included a smaller number of participants and therefore larger studies are required to replicate these initial promising findings.

As part of a larger study using mainly brain tissue samples, Dominy et al. [26] also used a limited number of matching CSF and saliva specimens in a pilot study of 10 probable AD patients with mild to moderate cognitive impairment (MMSE: 15–20, mean age: 59.6). These subjects met the criteria of MMSE scoring. Using a quantitative polymerase chain reaction (qPRC) assay, the authors quantified the load *Porphyromonas gingivalis (P. gingivalis)*, a keystone pathogen for the development of chronic periodontitis which was considered as a high-risk factor for AD and found that 7/10 CSF and all salivary samples were positive for the presence of *P. gingivalis.* According to the authors’ results, such a test for P. gingivalis may serve as a differential diagnostic marker.

Liu et al. [64] analyzed salivary samples from 39 AD (18 males, mean age: 64.3) and 39 healthy controls (21 males, mean age: 63.9). The diagnoses of probable or possible AD according to the NINCDS–ADRDA criteria [82]. Neuropsychological assessment was performed on all patients, including MMSE, neuropsychiatric inventory questionnaire (NPI), clinical dementia rating scale (CDR) and activity of daily living scale (ADL). MMSE was also conducted with healthy controls. Using 16S ribosomal RNA (rRNA) sequencing, they demonstrated significantly lower richness and diversity of microbiota in AD patients. Relative abundance of specific bacteria, such as *Moraxella*, *Leptotrichia* and *Sphaerochaeta*, was greatly increased whereas abundance of *Rothia* was significantly reduced. This study provided further support for the role of oral microbiome in AD and reported the altered composition of salivary microbiome, which may play role in the diagnosis of the disease.

### 2.8. Metabolites

Numerous studies have employed mass spectrometry techniques to develop a panel of distinguishing metabolites [65,66,67] or even specific biosensor assays [57] to detect individual diagnostic AD metabolites in saliva.

In the study of Lau et al. [57], which also studied the salivary levels of Aβ42, Τ-tau and *P*-tau with ELISA, a more specific biosensor, namely extended gate ion-sensitive field-effect transistor (EG-ISFET), was also used to study trehalose, a sugar molecule associated with the pathophysiology of AD. Trehalose levels were increased in AD patients (*n* = 18) in comparison to healthy controls (*n* = 18), leading the authors to conclude that such this test may serve as an alternative diagnostic tool for AD.

Huan et al. [65] used a liquid chromatography–mass spectrometry (LC-MS) technique to investigate potential metabolites that could serve as diagnostic AD biomarkers in saliva. The total sample number (*n =* 109) was divided into a discovery phase study (*n =* 82) and a validation phase study (*n =* 27). The discovery phase study included 22 AD (6 males, mean age: 77.1), 25 MCI (10 males, mean age: 70.4) and 35 healthy controls (13 males, mean age: 69.9), whereas the validation phase study included 7 AD (2 males, mean age: 70.1), 10 MCI (5 males, mean age: 71.5) and 10 controls (5 males, mean age: 71.4). Patients were required to have a diagnosis of AD based on DSM-IV criteria for dementia of the Alzheimer Type. Clinical assessments were performed as part of routine clinical evaluation, which included caregiver report of cognitive decline and impaired functional status, mental status evaluation of the patient (including the MMSE and Montreal Cognitive Assessment) and a physical and neurological examination. All patients had a medication review and had routine laboratory assessment for causes of dementia, including blood work and brain imaging according to Canadian Consensus Guidelines. For the MCI and control groups, an extensive and fully objective (non-clinical) classification was performed. After determining the top discriminating biomarkers, multi-metabolite panels distinguished AD from controls and MCI individuals with high diagnostic accuracies, achieving sensitivity ranging from 71–100% and a specificity of 80–100%. Saliva was characterized as a promising biofluid for both unbiased and targeted AD biomarker discovery.

Liang et al. [66] also used metabolomics to detect salivary biomarkers for early diagnosis of AD. This was one of the largest cohort studies with 256 AD samples (124 males, mean age: 78.6) and 218 age-matched healthy controls (102 males, mean age: 77.9). Diagnosis of AD was based on the NINCDS–ADRDA criteria whereas controls did not have any neurologic or cognitive disease. A number of different metabolites (sphinganine-1-phosphate, ornithine, phenyllactic acid, inosine, 3-dehydrocarnitine and hypoxanthine) were studied as discriminatory for AD with sphinganine-1-phosphate being able to achieve 99% sensitivity and 98% specificity.

Yilmaz et al. [67] used proton nuclear magnetic resonance (NMR) spectroscopy to study 9 AD (3 males, mean age: 85.0), 8 MCI (3 males, mean age: 83.0) and 12 healthy controls (4 males, mean age: 82.0). Similar to other metabolomics studies a panel of biomarkers was utilized to distinguish between the different cohorts with 91% sensitivity and 84% specificity.

### 2.9. Oxidative Stress Markers

Oxidative stress has been documented in tissues and biofluids of AD and MCI individuals. Su et al. [68] quantified protein carbonyl levels, a marker of oxidative stress, by ELISA in saliva samples of AD patients and found no significant difference between AD (*n* = 15) (10 males, mean age: 82.4) and MCI (*n* = 21) (10 males, mean age: 81.1) from controls (*n* = 30) (13 males, mean age: 69.2), however they reported a diurnal alteration in carbonyl levels. All AD subjects met the NINCDS–ADRDA criteria for probable AD. MCI individuals exhibited cognitive decline (usually memory) of at least 6 months duration that did not meet dementia criteria and scores of 0.5 on the Clinical dementia Rating scale. All subjects had the MMSE test. Controls had no memory complaints and scored ≥ on the MMSE.

Choromanska et al. [69] studied 80 individuals with different subtypes of dementia (24 AD (10 males, mean age: 77.9, mean MMSE score 13.1), 30 vascular dementia, 26 mixed dementia) and 80 healthy controls (25 males, mean age 80.1, mean MMSE score 27.4). The criteria for inclusion in the study group covered: cognitive impairment with undisturbed consciousness seen in the clinical picture and confirmed by the MMSE indicating a moderate dementia (score between 11 and 18 points on a 30-point scale), at least 6 months of positive history of cognitive impairment and no history of psychoactive substance abuse. Moreover, head CT scans of the patients excluded other diseases. The control group included people with MMSE >23 and normal results of complete blood count and biochemical blood tests. It was shown that in dementia patients the concentration of major salivary antioxidants changes and the level of oxidative damage to DNA, proteins and lipids is increased compared to healthy controls. Non-stimulated and stimulated salivary secretions were shown to be significantly reduced in dementia patients. The study’s overall conclusion was that dementia was associated with disturbed salivary redox homeostasis and impaired secretory function of the salivary glands.

## 3. Buccal Cells

### 3.1. Buccal Cells: General Considerations

Oral mucosa cytology samples can provide an almost non-invasive and relatively inexpensive alternative for AD diagnosis. Numerous studies have focused on the study of buccal cells as biomarkers in different neurological disorders since these cells (similarly to skin and brain cells) are derived from ectodermal tissue [30] and therefore are thought to be embryologically related to the central nervous system and share common AD-specific characteristics [83]. We would expect buccal cells to be advantageous in comparison to saliva specimens due to the increased cellular content [30]. Nevertheless, only a few studies were identified in the literature to study oral mucosal epithelial cells for AD diagnosis; herein, we present 9 of these studies (Table 2).

### 3.2. Buccal Cells: Specific Studies on AD

A study by Ozlece et al. [70] utilized microscopic analysis of oral mucosa to perform cytological and cytometric analysis. The included cohort consisted of 29 AD patients (16 males, mean age: 78.9), 30 patients with Parkinson’s disease and 30 healthy individuals (15 males, mean age: 79.5). AD diagnosis was given according to the NINCDS–ADRDA criteria. The MMSE and Clinical dementia Scale were used to assess AD patients. The authors evaluated the nuclear and cytoplasmic volumes of buccal cells derived from the different groups and did not find significant differences.

Mathur et al. [71] utilized three-dimensional imaging of telomeres to differentiate 41 AD patients (19 males, mean age: 75.8) from 41 healthy volunteers (19 males, mean age: 74.3). The NINCDS–ADRDA criteria were used for AD diagnosis. AD patients were categorized in mild, moderate or severe AD based on their regular clinic visits and their scores on the MoCA and the MMSE. The control group consisted of age- and gender-matched cognitively normal caregivers. All AD patients, from mild to severe cases, had significantly different telomere profiles. An increase in telomer number and aggregation as well as a decrease in the telomere length were shown to differentiate the different stages of the disease (from normal to severe AD).

Two different studies by Francois et al. [72,73] utilized automated laser-scanning cytometry as the experimental approach to analyze buccal cells and identify altered parameters in AD patients. The earlier study [72] recruited 13 AD (2 males, mean age: 77.7), 13 MCI (2 males, mean age: 75.3) and 26 healthy (4 males, mean age: 76.1) controls and showed increased DNA content and increased abnormal shape in MCI and AD when compared to controls, as well as a decrease of neural lipid content in MCI. Diagnosis of both MCI and AD was according to the criteria outlined by the NINCDS–ADRDA while controls were not clinically diagnosed with MCI or AD. The subsequent study by the same group [73] attempted to simultaneously measure cell types, nuclear DNA content and aneuploidy, neutral lipid content, putative Tau and Aβ in buccal cells, some of which showed some contradictory results with their earlier study. In this latter study, 20 AD, 20 MCI and 20 healthy controls were included, with results showing no change in the DNA content, aneuploidy, neutral lipids and Tau, whereas a significant decrease in Tau in basal and karyolytic cells when compared to differentiated buccal cells. Aβ was found significantly higher in the AD group when compared to controls.

Thomas et al. conducted numerous studies towards the development of AD biomarkers [74,75,76]. A buccal cytome assay was used in one of these studies [74] to measure ratios of buccal cell populations and micronuclei in 54 AD patients (16 males, mean age: 76.9) compared to 26 age- and gender-matched controls (11 males, mean age: 68.7). Diagnosis was based on the NINCDS–ADRDA criteria. Frequencies of basal cells (*p* < 0.0001), condensed chromatin cells (*p* < 0.0001) and karyorrhectic cells (*p* < 0.0001) were found to be significantly lower in AD. The study’s authors concluded that buccal cytome biomarkers may be associated with AD, but also urged for larger studies to replicate these pilot results. A second study from the same group [75], measured the incidence of chromosome 17 and 21 aneuploidy, which have been suggested to contribute to the etiology of AD. Including 54 AD patient and 56 healthy controls, they reported a 1.5-fold increase in trisomy 21 and a 1.2-fold increase in trisomy 17 in buccal cells derived from AD. However, when they compared aneuploidy rates in the nuclei of hippocampus brain cells, they found no significant differences between the groups, leading to the suggestion that aneuploidy events may be influenced by genetic factors that may predispose to AD, but they are unlikely to be a primary cause of AD brain pathology. Another study from Thomas et al. [76] studied telomere length, which has been associated with aging and degeneration, using a real-time PCR method in 54 AD and 56 controls. They observed a significantly lower telomere length in white blood cells and buccal cells in AD, however telomere length was significantly higher in hippocampus cells of AD, suggesting important differences in telomere maintenance depending on the sample type.

Hattori et al. [77] used oral epithelial cells exfoliated from 34 AD (11 males, mean age: 74.8), 29 vascular dementia and 67 healthy controls (29 males, mean age: 57.2) as well as CSF samples. All patients fit the diagnosis of probable AD based on the NINCDS–ADRDA criteria. The healthy control group was age-matched and showed no neurological symptoms, signs or dementia. Using western blot and ELISA they determined the Tau protein levels, which showed a significant positive correlation with levels in the CSF. Patients with AD had significantly higher levels of Tau than the patients with vascular dementia and the controls. AD patients with a younger age at onset showed a higher level of Tau than patients with later age at onset.

Finally, Garcia et al. [78] used super-resolution microscopy to study the structure of DNA in buccal cells. Quantitation of the super-resolution DNA structure in 37 AD patients and 37 controls, revealed that the structure of individuals with AD significantly differed from controls with an overall increase in the measured DNA-free/poor spaces, which represents a significant increase in the interchromatin compartment. AD diagnosis was made according to NINCDS–ADRDA criteria and patients were classed as mild, moderate or severe AD based on their regular visits and their scores on the MoCA and the MMSE.

## 4. Conclusions

With a few exceptions, most studies utilizing oral samples, either saliva or buccal cells, for AD diagnosis, screening or disease monitoring have been performed in small-numbered cohorts, which can be explained by the fact that this is a new, underexplored field. Low-powered studies and the emergence of contradictory results between different publications and research groups, render it difficult to come to a definitive conclusion on whether salivary markers can accurately detect AD. Currently statistically insignificant studies may reach statistical significance, and vice versa, in future larger-cohort studies. It should also be emphasized that the various studies have utilized different diagnostic criteria, based on clinical diagnosis alone or confirmation with molecular and imaging biomarkers. The cohorts with a standard clinical diagnosis run the risk of including other subtypes of dementia, which may confound the results.

This review has reported extensively on studies that have used different experimental approaches, ranging from molecular assays to metabolomics and cytological assessment, as well as different biomarkers, ranging from Aβ and tau proteins to oral microbiome, toward AD investigation. Studies were included irrespective of their results, demonstrating promising or not-promising biomarkers and their final outcomes were fully described. Both advantages and disadvantages of salivary specimens were discussed.

Further large-cohort studies are necessary to determine the use of these non-invasive oral samples as a research and/or clinical tool for AD. There is need for independent studies from different research groups to validate results and allow standardization of saliva collection, processing and storage.

Taking into consideration the plethora of different markers that have been so far suggested as promising screening/diagnostic markers for AD, it is obvious that there are many causes with one endpoint. It is therefore expected that a multiple panel marker approach will reveal more information rather than individual biomolecules. Such an approach could be used as a “first-line” screening tool to identify individuals who may need further referral for imaging or CSF testing.

## Figures and Tables

**Table 1:**
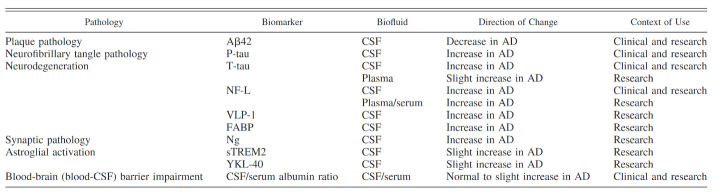
Replicated biomarkers detected in biofluids (cerebrospinal fluid and blood plasma/serum) of Alzheimer’s disease patients. Adapted from Zetterberg, 2017 [25].

Abbreviations: AD, Alzheimer’s disease; Aβ42, the 42 amino acid form of amyloid-β; P-tau, phosphorylated tau; T-tau, total tau; NF-L, neurofilament light; VLP-1, visinin-like protein 1; FABP, fatty acid-binding protein; Ng, neurogranin; sTREM2; secreted triggering receptor expressed on myeloid cells 2; CSF, cerebrospinal fluid.

**Table 2:** Comprehensive table of our literature review. Potential biomarkers, type of non-invasive sample used (saliva or buccal cells), experimental techniques, number of participants and the main findings for each separate study are all reported.

Type of Sample	Potential Biomarker	Experimental Technique	Cohort (*n*)	Main Findings	References
Saliva					
	**Aβ42**	Sandwich ELISA	AD: 15 Healthy controls: 7	Increased	Sabbagh et al. [52]
		Sandwich ELISA	AD: 23 Healthy controls: 31	Increased	McGeer et al. [53]
		Sandwich ELISA	AD: 7 Pre-AD: 3Healthy controls: 26	Increased	Lee et al. [54]
		Sandwich ELISA	AD: 20 Healthy controls: 20	Not detected	Lau et al. [57]
		Sandwich ELISA	AD: 70 Healthy controls: 56	Increased	Bermejo-Pareja et al. [55]
		Nanobead ELISA	AD: 28 Healthy controls: 17	Increased	Kim et al. [56]
		Luminex ELISA	AD: 21 Healthy controls: 38	Not detected	Shi et al. [42]
	**Aβ40**	Sandwich ELISA	AD: 70 Healthy controls: 56	No change	Bermejo-Pareja et al. [55]
		Nanobead ELISA	AD: 28 Healthy controls: 17	No change	Kim et al. [56]
	**T-tau**	Sandwich ELISA	AD: 20 Healthy controls: 20	No change	Lau et al. [57]
		Luminex ELISA	AD: 21 Healthy controls: 38	No change	Shi et al. [42]
		SIMOA	AD: 53 MCI: 68 Healthy controls: 160	No change	Ashton et al. [58]
	***P*-tau**	Sandwich ELISA	AD: 20 Healthy controls: 20	No change	Lau et al. [57]
		Luminex ELISA	AD: 21 Healthy controls: 38	No change	Shi et al. [42]
	***P*-tau/T-tau**	Luminex ELISA	AD: 21 Healthy controls: 38	Increased	Shi et al. [42]
		Western blot	AD: 87 MCI: 55 Healthy controls: 167 (used in two consequent studies)	Increased	Pekeles et al. [59]
	**Lactoferrin**	Mass spectrometry and sandwich ELISA	AD: 116 MCI: 59 Healthy controls: 131 (used in two studies: discovery and validation)	Decreased	Carro et al. [60]
	**Acetylcholinesterase**	Ellman’s colorimetric method	AD: 36 Healthy controls: 11	Decreased	Sayer et al. [61]
		Ellman’s colorimetric method	AD: 15 Healthy controls: 13	No change	Boston et al. [62]
		Ellman’s colorimetric method	AD: 15 Healthy controls: 15	No change	Bakhtiari et al. [63]
	**Oral Microbiome (Porphyromonas Gingivalis)**	qPCR	AD: 10	Detection of *P. Gingivalis* DNA in 10/10 saliva samples	Dominy et al. [26]
	**Oral Microbiome**	16S rRNA sequencing	AD: 39 Healthy controls: 39	Significantly lower richness and diversity of microbiota detected in AD than healthy controls. Relative abundance of *Moraxella*, *Leptotrichia* and *Sphaerochaeta* in the saliva of AD was greatly increased, whereas that of *Rothia* was significantly reduced	Liu et al. [64]
	**Metabolites: trehalose**	EG-IDFET biosensor	AD: 20 Healthy controls: 20	Increased	Lau et al. [57]
	**Metabolites: multiple marker panel**	LC-MS	AD: 29 MCI: 35 Healthy controls: 45 (used in two studies: discovery and validation)	High diagnostic accuracies (sensitivity: 71–100% and specificity: 80–100%)	Huan et al. [65]
	**Metabolites: multiple marker panel**	FUPLC-MS	AD: 256 Healthy controls: 218	High diagnostic accuracies (sensitivity: 82–99% and specificity: 91–98%)	Liang et al. [66]
	**Metabolites: multiple marker panel**	NMR spectroscopy	AD: 9 MCI: 8 Healthy controls: 12	High diagnostic accuracies (sensitivity: 91% and specificity: 84%)	Yilmaz et al. [67]
	**Oxidative stress markers (protein carbonyl levels)**	ELISA	AD: 15 MCI: 21 Healthy controls: 30	No change	Su et al. [68]
	**Salivary flow, antioxidants and oxidative damage products**	Total protein content, colorimetry, fluorimetry	AD: 24 Healthy controls: 80	Decreased salivary flow; increased total protein concentration; decreased antioxidant properties; increased levels of oxidative damage to DNA, protein and lipids	Choromanska et al. [69]
**Buccal cells**	**Cytological & cytometric analysis**	Microscopic analysis	AD: 29 Healthy controls: 30	No changes in the cytoplasmic and nuclear volumes	Ozlece et al. [70]
	**Imaging of telomeres**	3D telomeric analysis	AD: 41 Healthy controls: 41	Significantly different 3D telomere profiles. Increased telomere number and aggregation and decrease in telomere length differentiated from normal to severe AD	Mathur et al. [71]
	**Altered cytological parameters**	Automated laser-scanning cytometry	AD: 13 MCI: 13 Healthy controls: 26	Increased DNA content. Increased abnormal nuclear shape. Decreased neutral lipid content in MCI	Francois et al. [72]
	**Multiparameter analysis**	Automated laser-scanning cytometry	AD: 20 MCI: 20 Healthy controls: 20	No change in DNA content, aneuploidy, neutral lipids, tau. Lower tau in basal and karyolytic cells versus differentiated cells. Increased Aβ	Francois et al. [73]
	**Buccal micronucleus cytome markers**	Buccal cytome assay	AD: 54 Healthy controls: 26	Decreased frequencies of basal, condensed chromatin and karyorrhectic cells	Thomas et al. [74]
	**Incidence of chromosome 17 & 21 aneuploidy**	FISH & fluorescently labeled DNA probes	AD: 54 Healthy controls: 56	Increased	Thomas et al. [75]
	**Telomere length**	Real time PCR	AD: 54 Healthy controls: 56	Decreased	Thomas et al. [76]
	**T-tau**	Western blot ELISA	AD: 34 Healthy controls: 67	Increased. Correlated with observed increase in CSF	Hattori et al. [77]
	**DNA structure**	Super-resolution microscopy	AD: 37 Healthy controls: 37	Increase of the measured DNA-free/poor spaces (i.e., increase in interchromatin compartment)	Garcia et al. [78]

Abbreviations: AD: Alzheimer’s disease; Aβ: amyloid beta; CSF: cerebrospinal fluid; EG-ISFET: extended gate ion-sensitive field-effect transistor; ELISA: enzyme-linked immunosorbent assay; FISH: fluorescent in situ hybridization; FUPLC-MS: faster ultra-performance liquid; chromatography-mass spectrometry; NMR: nuclear magnetic resonance; LC-MS: liquid chromatography-mass spectrometry; MCI: mild cognitive impairment; qPCR; quantitative polymerase chain reaction; rRNA: ribosomal RNA; SIMOA: single molecule array.
